# Complete genome sequence of a *Clostridioides difficile* cryptic C-III strain isolated from horse feces

**DOI:** 10.1128/MRA.00781-23

**Published:** 2023-11-01

**Authors:** Miriam Antonia Schüler, Rolf Daniel, Anja Poehlein

**Affiliations:** 1 Genomic and Applied Microbiology and Göttingen Genomics Laboratory, Institute of Microbiology and Genetics, Georg-August University of Göttingen, Göttingen, Germany; The University of Arizona, Tucson, Arizona, USA

**Keywords:** *Clostridioides difficile*, cryptic C-III strain, horse feces, non-toxigenic strain

## Abstract

We provide the complete genome of a non-toxigenic *Clostridioides difficile* strain isolated from horse feces. The strain represents a sub-cluster in the cryptic clade C-III. The genome consists of one chromosome (4,144,784 bp) and one plasmid (10,144 bp) and encodes 3,798 putative genes.

## ANNOUNCEMENT

The worldwide pathogen *Clostridioides difficile* covers at least five phylogenetic clades plus the cryptic clades C-I to C-V. We provide the complete genome of the C-III *C. difficile* strain MA_2, which was isolated from horse feces based on Dharmasena and Jiang (1). All enrichment and cultivation steps were performed under anaerobic conditions with incubation at 37°C. Twenty-five grams of sample were suspended in 100 mL phosphate buffered saline (PBS) (137 mM NaCl, 2.7 mM KCl, 10 mM Na_2_HPO_4_, 1.8 mM KH_2_PO_4_, pH 7.4), solid particles filtered out, and cells washed repeatedly by centrifugation (4,000 × *g*, 10 minutes) before cultivation in 20 mL BHIB-YE-CYS-MN-T medium (1) for 7 days. One milliliter of enriched culture was heat-shocked (60°C, 25 minutes), cells pelleted, suspended in 100 µL PBS, plated on CDA-CYS-H-MN-T plates (1), and cultivated for 2 days. Single colonies were cultivated in BHIS medium (Brain Heart Infusion Broth, 0.5% yeast extract, 0.05% L-cysteine), and 2 µL were used as input for 16S rRNA gene PCR, using Phusion High-Fidelity polymerase (Thermo Fisher Scientific, Waltham, MA, USA) with GC buffer and PCR components according to protocol and 0.2 µM of primers (08 f: 5′-AGAGTTTGATCCTGGC-3′, 1504 r: 5′-TACCTTGTTACGACTT-3′). PCR cycling comprised 98°C for 6 minutes, 28 cycles of 10 s at 98°C, 15 s at 55°C, and 40 s at 72°C, followed by 5 minutes at 72°C. Purified PCR products were Sanger sequenced (Microsynth Seqlab GmbH, Göttingen, Germany). The best sequence match was *C. difficile* strain ZZV14-6009 16S rRNA gene (KX792125.1; query coverage 99%, percentage of identity 99.93%) according to BLASTn (2).

The previous culture was inoculated in BHIS medium and cultivated as stated above before using 2 mL for DNA isolation with the MasterPure Gram Positive DNA Purification kit as recommended by the manufacturer (Epicentre, Madison, WI, USA). Illumina Nextera XT DNA libraries were sequenced on a MiSeq instrument (v3 chemistry; 2 × 300 bp, 600 cycles) as recommended by the manufacturer (Illumina, San Diego, CA, USA). For Nanopore sequencing, genomic DNA was isolated as described before from 4 mL of another fresh overnight culture cultivated as stated above. Libraries were prepared without DNA size selection using the ligation sequencing kit 1D (SQK-LSK109) and the native barcode expansion kit (EXP-NBD104) according to the manufacturer (Oxford Nanopore Technologies, Oxford, UK), and sequencing was performed for 72 h using a SpotON flow cell Mk I (R9.4.0) and MinKNOW software v19.12.5, with integrated Guppy v3.2.10 for base calling (fast mode) and demultiplexing. Short reads were processed with fastp v0.23.3 (3) and trimmed with Trimmomatic v0.39 (4). Long reads were trimmed using Porechop v0.2.4 (https://github.com/rrwick/Porechop), filtered with Filtlong v0.2.1 (https://github.com/rrwick/Filtlong), and assembled with Flye v2.9.2 (5). The assembly was polished with short reads using BWA v0.7.17 (r1188) (6) and Polypolish v0.5.0 (7), resulting in one complete chromosome (4,144,784 bp) and plasmid (10,144 bp). Circlator v1.5.5 was used for start position adjustment (8). Prokka v1.14.5 (9) was used for annotation (selenoproteins were curated manually), PubMLST (10) for sequence type assessment, and toxin genes were detected with virulence factor database (VFDB) (11). All software was used with default settings unless otherwise stated. Sequencing statistics and genome features are listed in Table 1.

**TABLE 1:** Sequencing statistics and genome features of strain MA_2

Sequencing statistics		Genome features	
No. of short reads	3,026,482	Length chromosome (bp)	4,144,784
No. of long reads	18,911	Length plasmid (bp)	10,144
Long-read *N*_50_ (bp)	29,017	GC content (%)	28.68
Coverage short reads	166	No. of tRNAs	92
Coverage long reads	356	No. of tmRNA	1
		No. of rRNAs	35
		No. of CRISPRs	5
		No. of CDSs	3,798
		No. of curated selenoproteins	3
		Sequence type	340
		Toxin gene profile	A^─^ B^─^ CDT^─^

**Fig 1:**
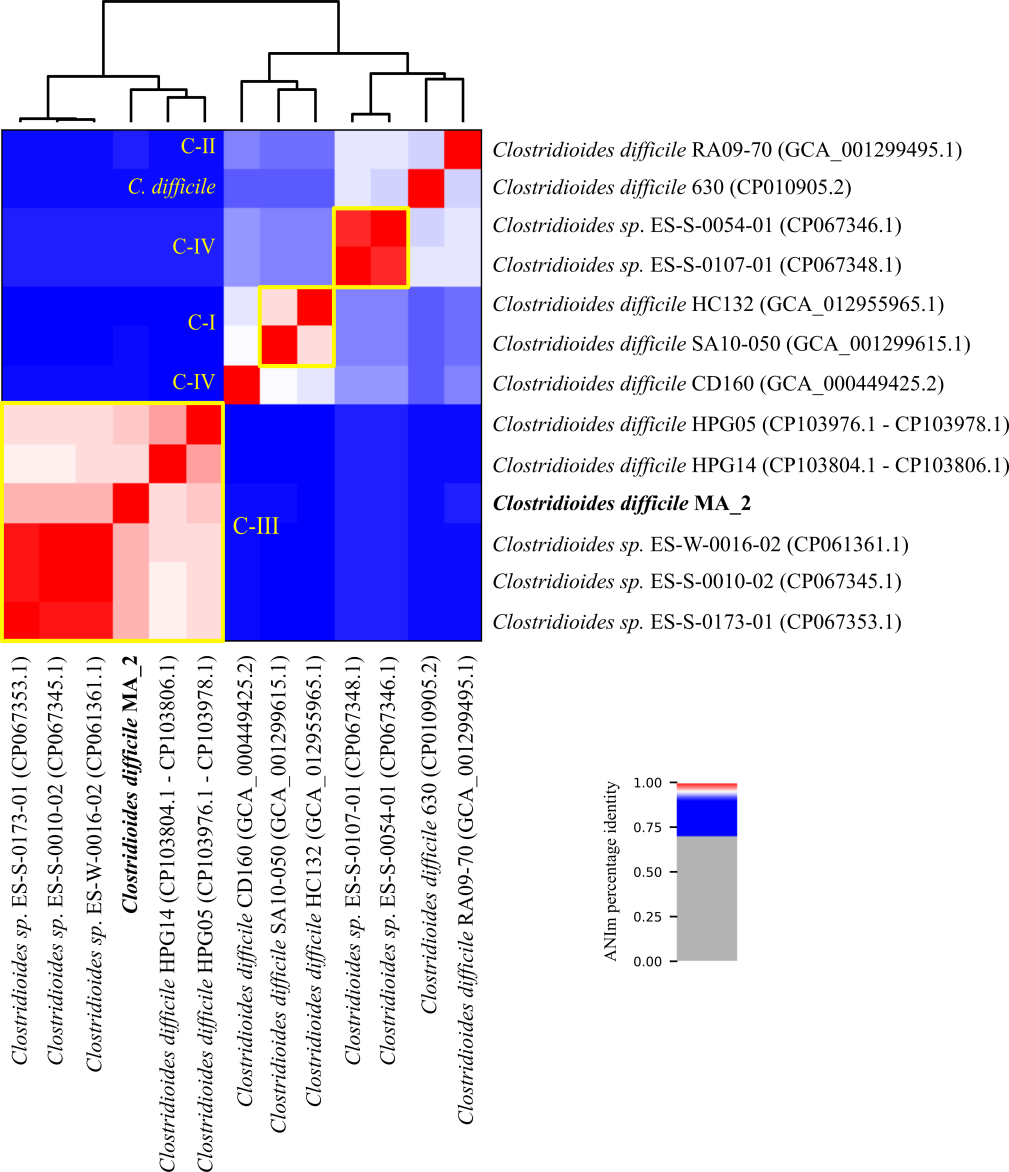
ANIm analysis of strain *C. difficile* MA_2. Representative genomes of cryptic clades C-I to C-V (14, 15) and of *C. difficile* strain 630 representing clades C-I to C-V were included in ANIm calculations against *C. difficile* MA_2 (bold). GenBank accession numbers are provided in parenthesis, and clade affiliation is stated in yellow.

An average nucleotide identity (ANI) analysis with pyani v0.2.12 (12) using MUMmer3 (13) (ANIm) was performed for clade assignment. Strain MA_2 clustered with representative strains of cryptic clades C-I to C-V (14, 15) (Fig. 1).

## Data Availability

The complete genome sequence is deposited under GenBank accession numbers CP129431.1 (chromosome) and CP129432.1 (plasmid), and the 16S rRNA gene sequence is deposited under OR144343.1. Corresponding raw reads are accessible in the NCBI Sequence Read Archive (SRA) under accession numbers SRR24958190 (Nanopore reads) and SRR24958186 (Illumina reads).
